# Supplementary material for: Key determinants of global land-use projections
Source: Nat Commun. 2019 May 15;10:2166. doi: 10.1038/s41467-019-09945-w (PMC6520344; doi:10.1038/s41467-019-09945-w)
Supplement: Supplementary file 1 — Supplementary Information [file 41467_2019_9945_MOESM1_ESM.pdf]

Supplementary Information:

Key determinants of global land-use projections

Stehfest et al.

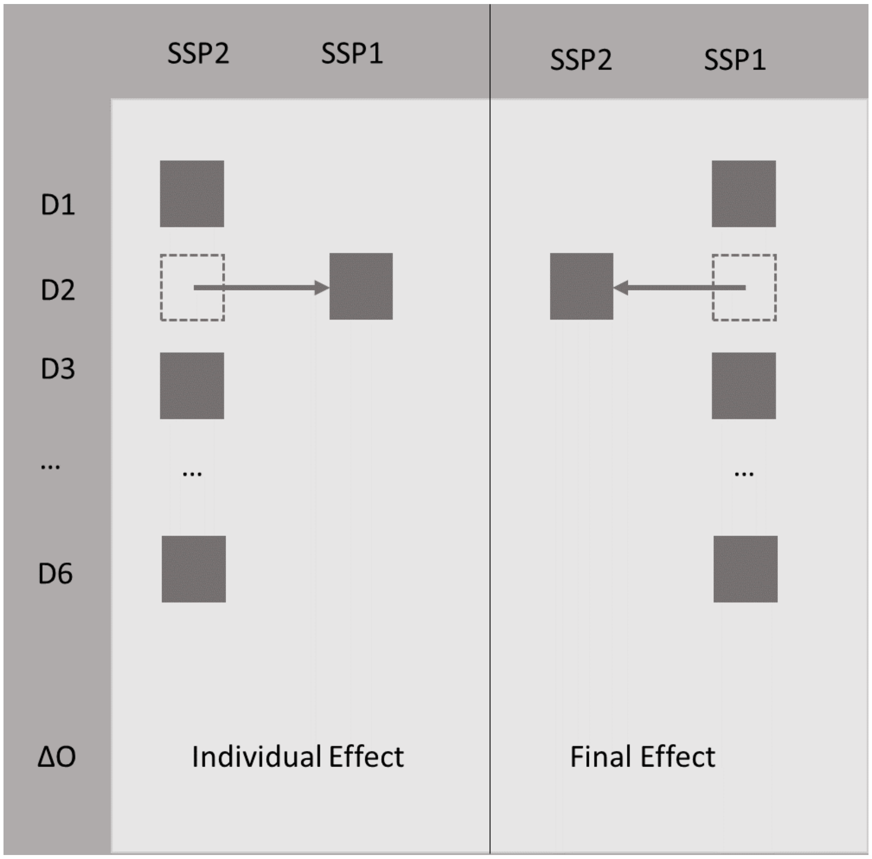

Supplementary Figure 1: Schematic representation of the sensitivity methodology. Example for SSP1: For the individual effect, one driver (D2), e.g. GDPpc, among all six considered is moved from the reference level of scenario SSP2 to the alternative level of scenario SSP1. The difference in output (ΔO), e.g. cropland, yields the individual effect of D2 on the output. For the final effect, all factors but D2 are moved to the alternative level from the reference level. With a change in sign, the difference in outputs yields the final effect. The difference between total and individual effects are the interactions. Adopted from Marangoni, et al. [1].

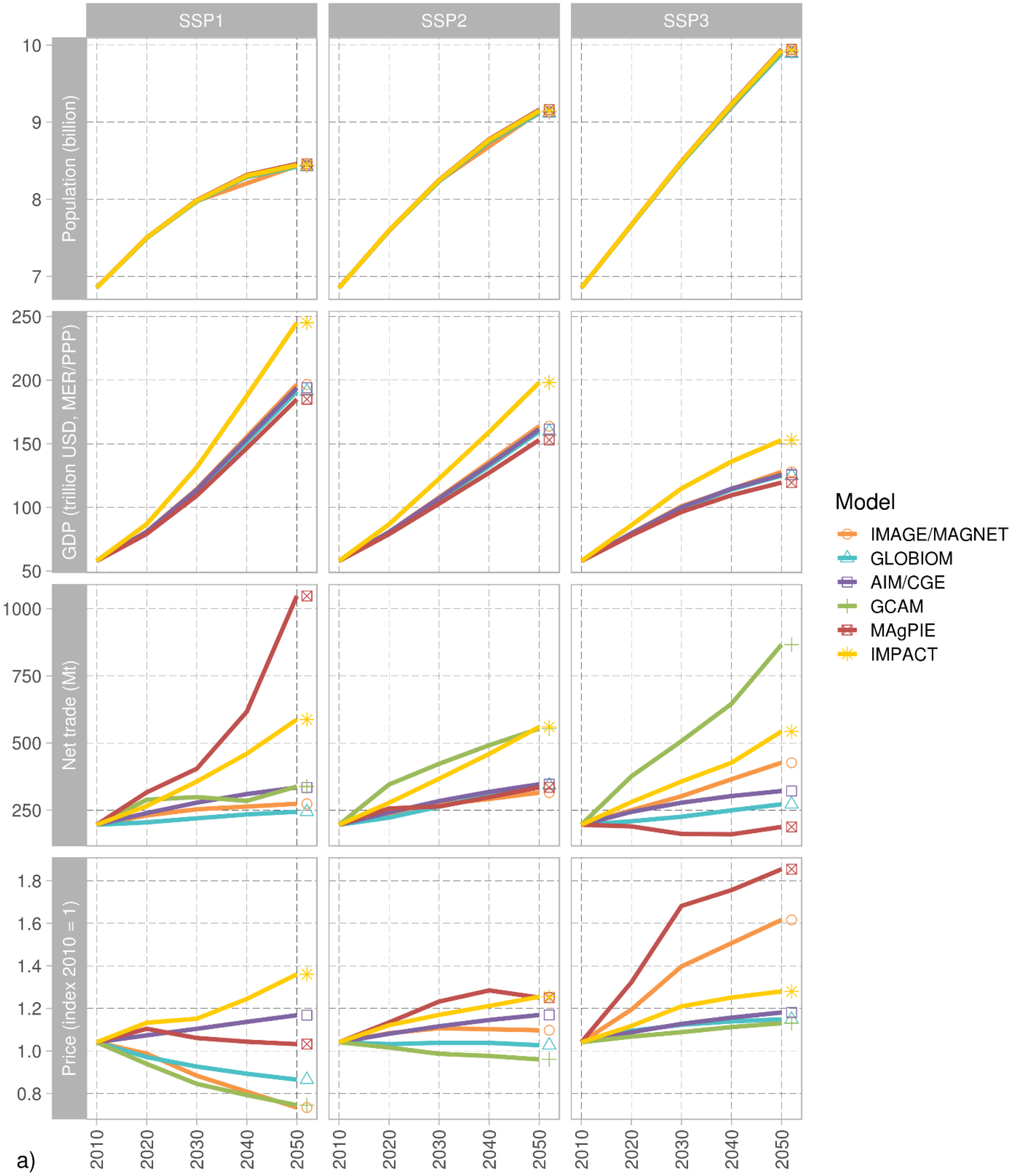

SSP1
SSP2
SSP3
Population (billion)
GDP (trillion USD, MER/PPP)
Net trade (Mt)
Price (index 2010 = 1)
Model
IMAGE/MAGNET
GLOBIOM
AIM/CGE
GCAM
MAgPIE
IMPACT

a)

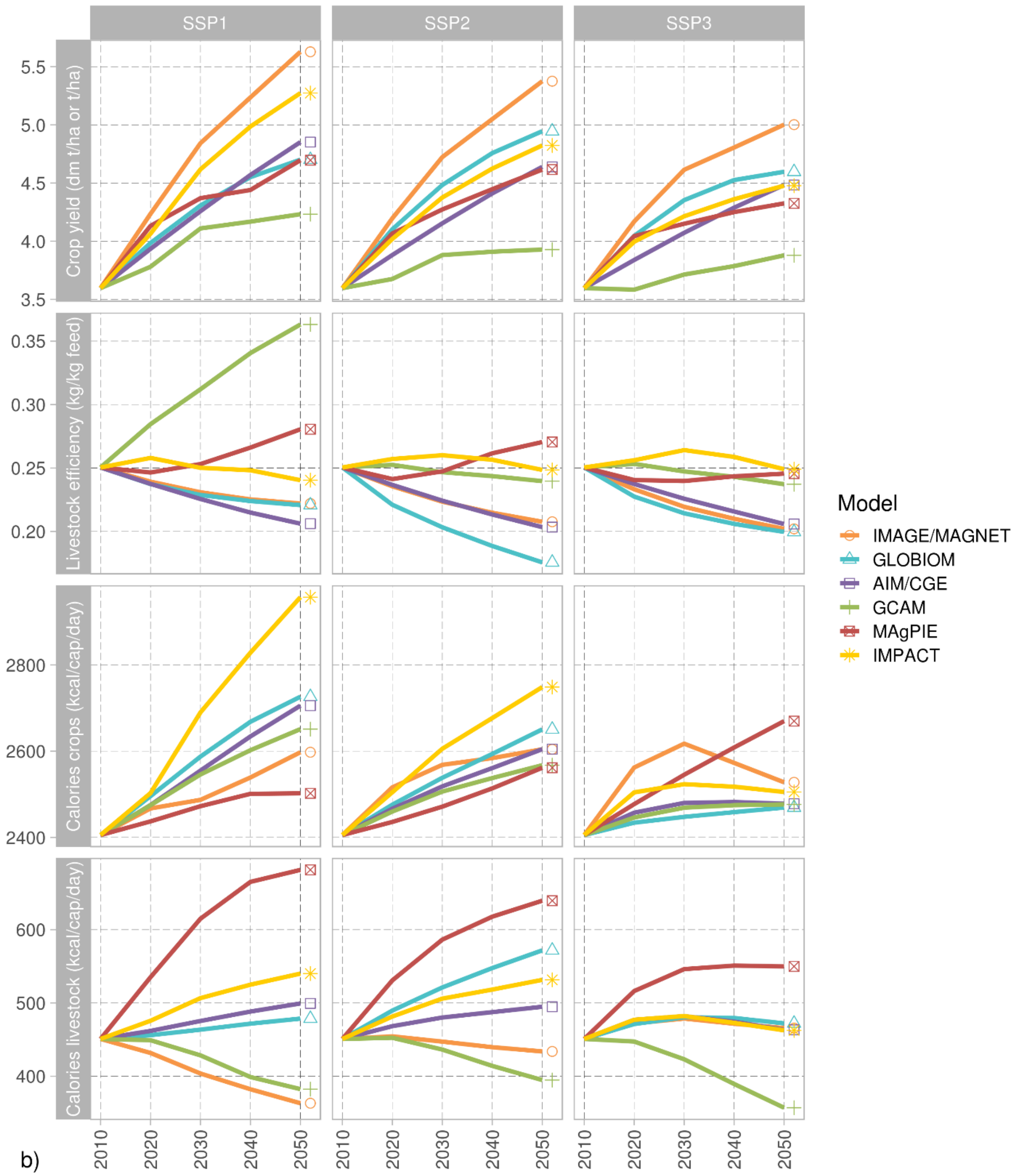

b)

Supplementary Figure 2: Additional characteristics of global agriculture and land use in SSP1, SSP2 and SSP3. Population, GDP per capita, agricultural price and trade volumes (a), crop yields, livestock system efficiency, crop consumption, livestock consumption (b). Results are scaled to one common starting values in 2010 (where possible from FAO, otherwise 2010 value in IMAGE model).

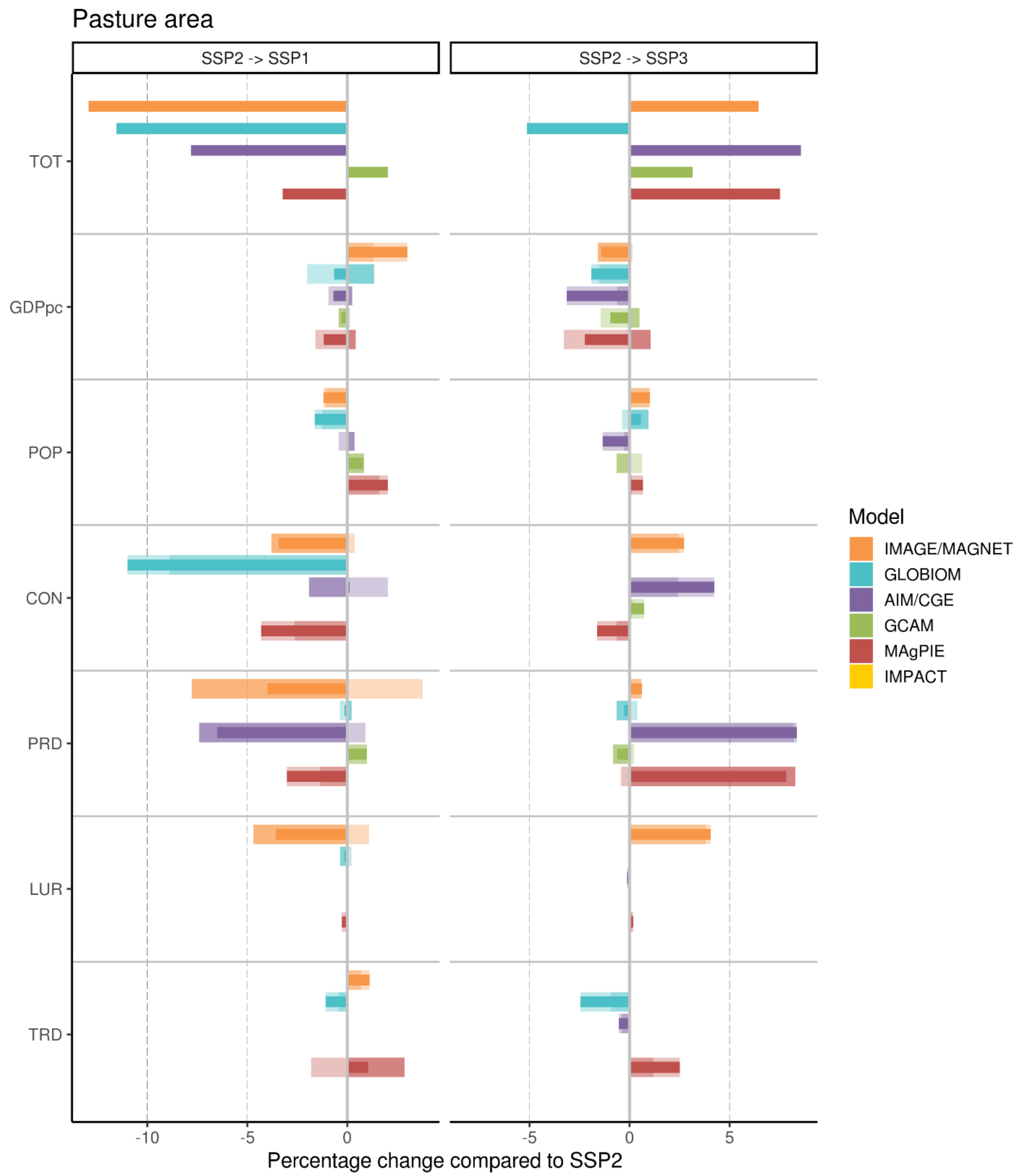

Supplementary Figure 3: Sensitivity of pasture area in SSP1 and SSP3 outcomes to groups of drivers. Changes refer to global pasture area in 2050 when moving from SSP2 to either SSP1 (left) or SSP3 (right). Individual effects (wide, light colored bars), final effects (thin, dark colored bars) and interaction effects (wide, very light colored bars) are shown for each of the six driver groups (POP, GDPpc, LUR, PRD, CON, TRD), together with the total difference between SSP1 or SSP3, respectively, to SSP2 (TOT). The IMPACT model does not include grassland areas.

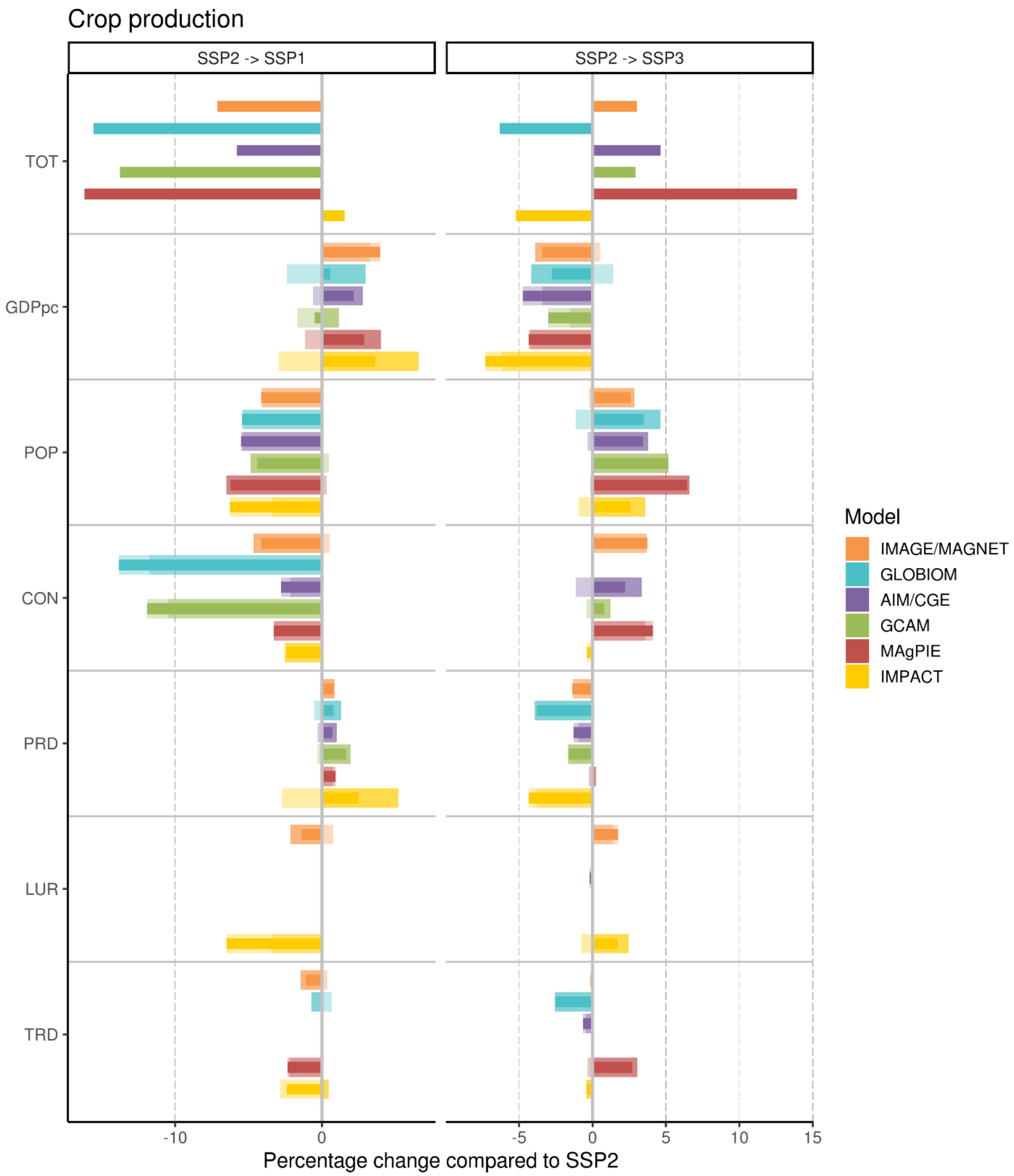

Supplementary Figure 4: Sensitivity of crop production in SSP1 and SSP3 to groups of drivers. Changes refer to production change in 2050 when moving from SSP2 to either SSP1 (left) or SSP3 (right). Individual effects (wide, light colored bars), final effects (thin, dark colored bars) and interaction effects (wide, very light colored bars) are shown for each of the six driver groups (POP, GDP, LUR, PRD, CON, TRD), together with the total difference between SSP1 and SSP3, respectively, and SSP2 (TOT).

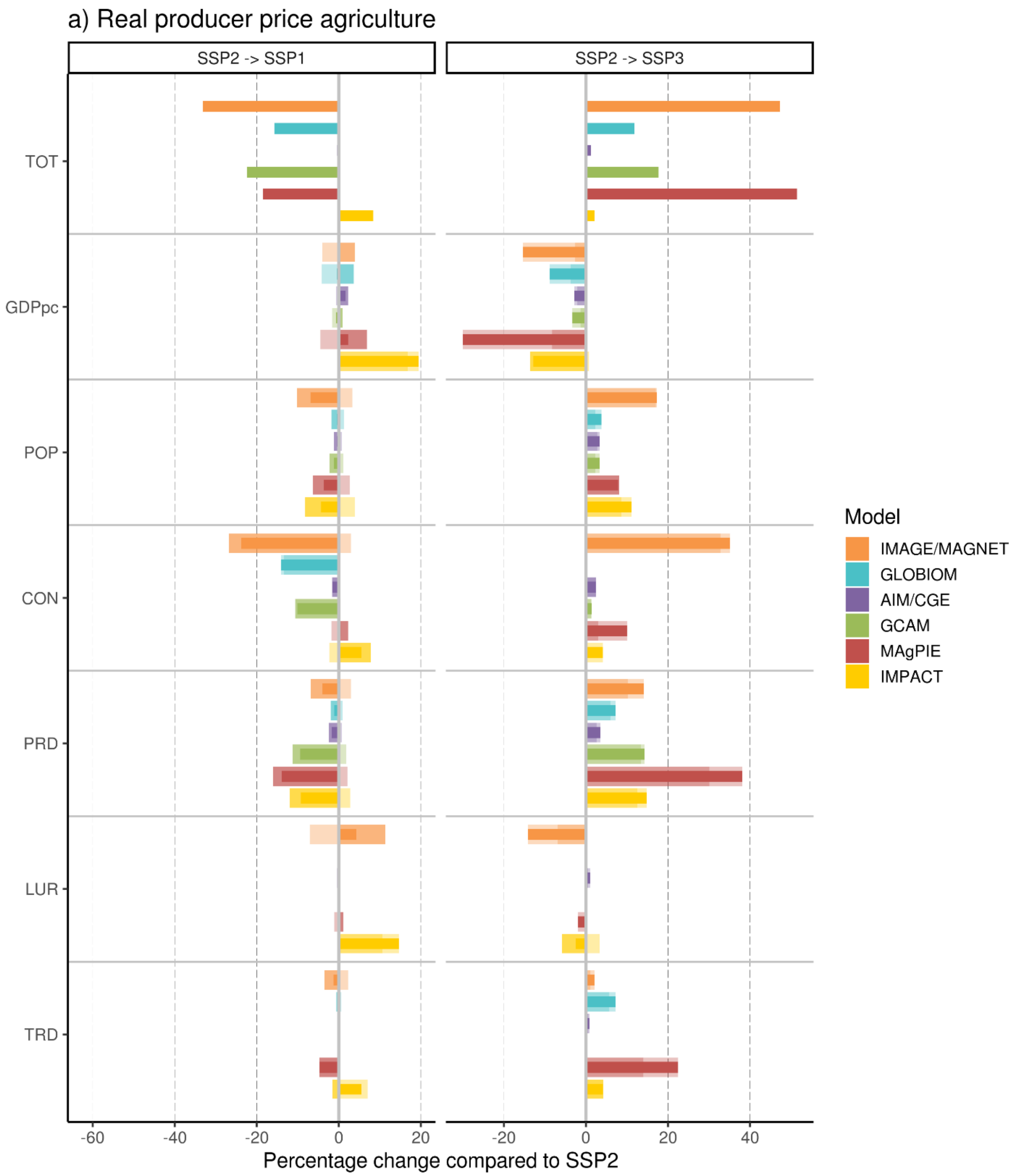

a) Real producer price agriculture
SSP2 -> SSP1
SSP2 -> SSP3
TOT
GDPpc
POP
CON
PRD
LUR
TRD
-60
-40
-20
0
20
-20
0
20
40
Percentage change compared to SSP2
Model
IMAGE/MAGNET
GLOBIOM
AIM/CGE
GCAM
MAgPIE
IMPACT

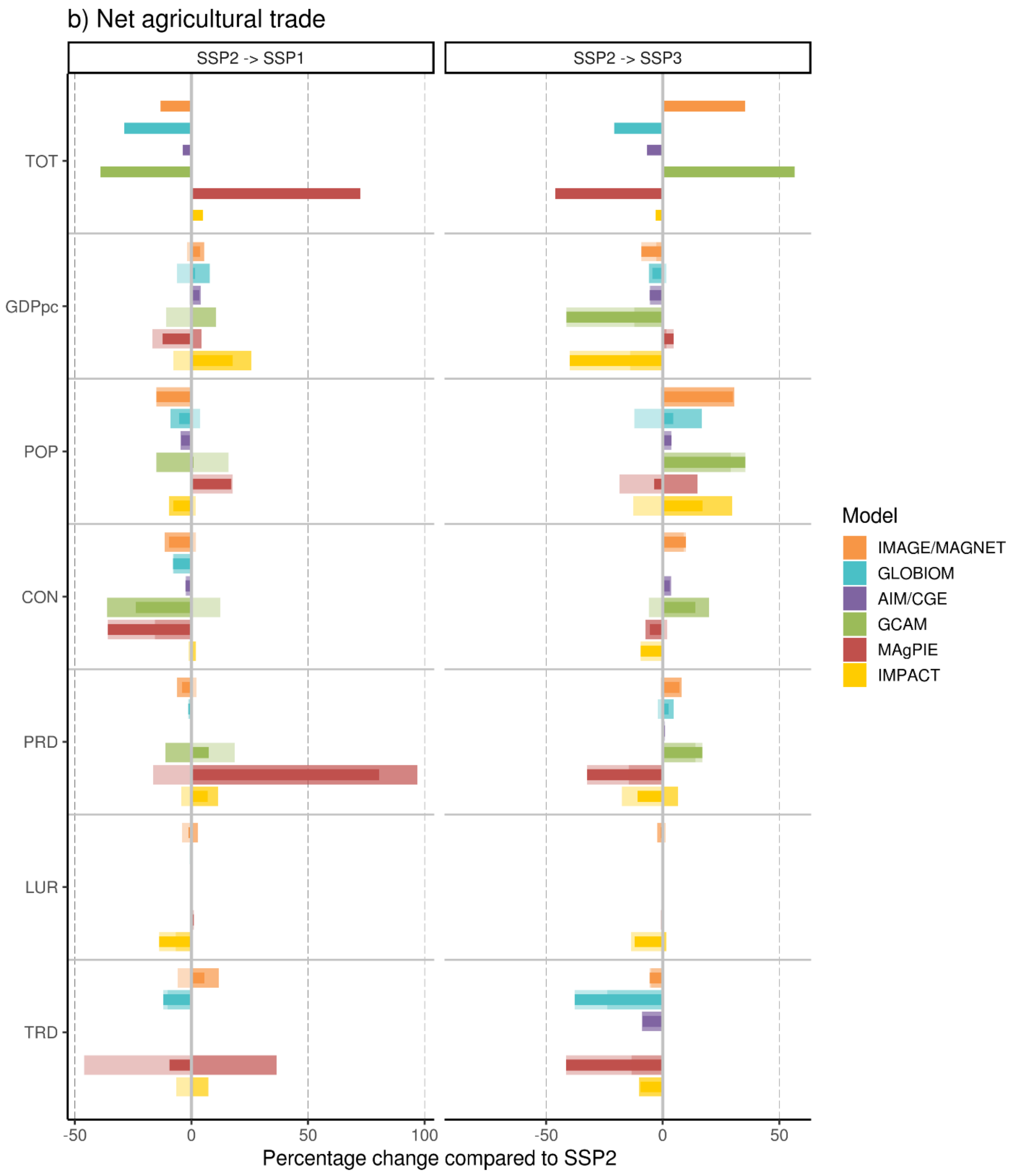

b) Net agricultural trade
SSP2 -> SSP1
SSP2 -> SSP3
TOT
GDPpc
POP
CON
PRD
LUR
TRD
-50
0
50
100
-50
0
50
Percentage change compared to SSP2
Model
IMAGE/MAGNET
GLOBIOM
AIM/CGE
GCAM
MAgPIE
IMPACT

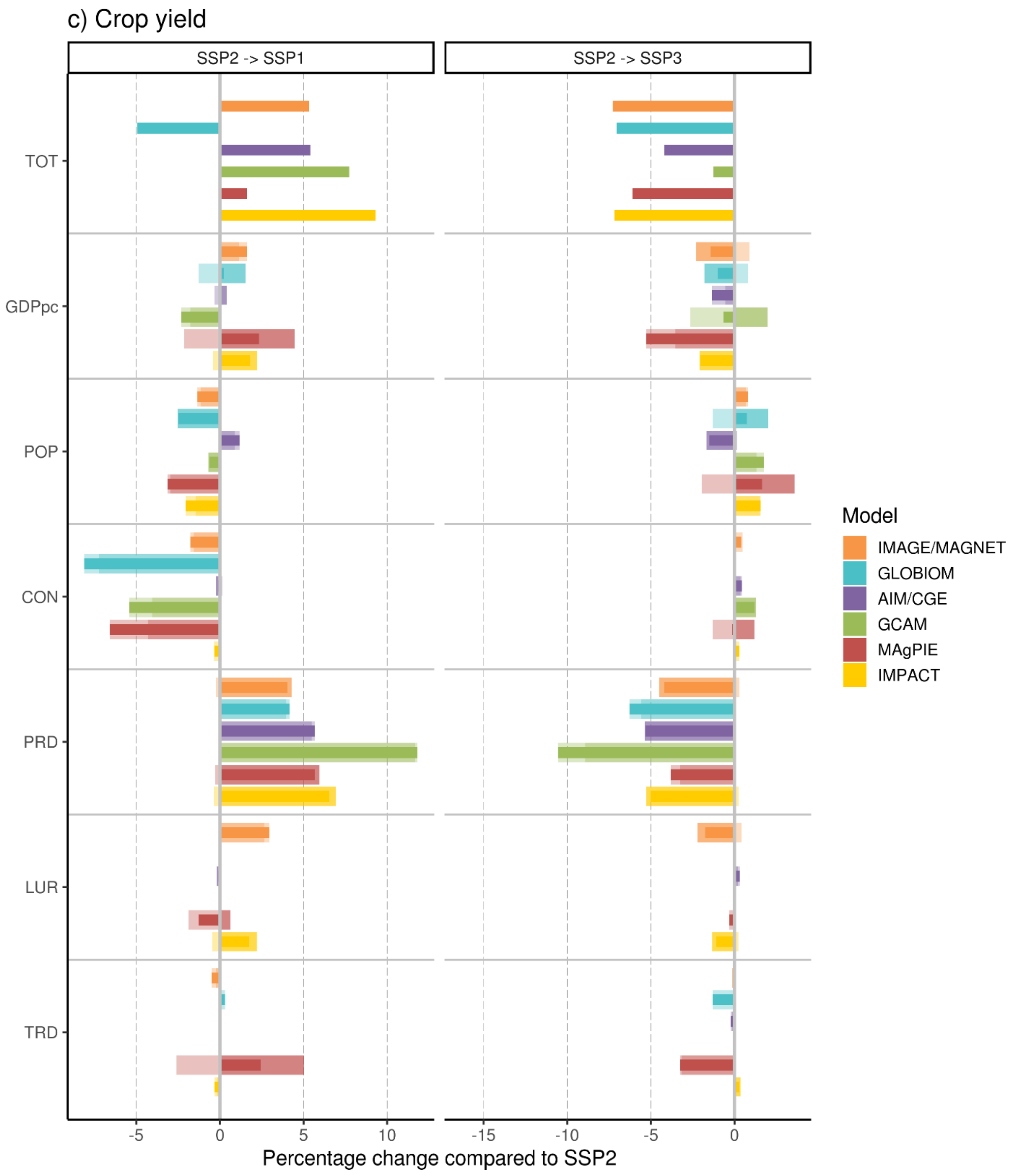
c) Crop yield
SSP2 -> SSP1
SSP2 -> SSP3
TOT
GDPpc
POP
CON
PRD
LUR
TRD
-5
0
5
10
-15
-10
-5
0
Percentage change compared to SSP2
Model
IMAGE/MAGNET
GLOBIOM
AIM/CGE
GCAM
MAgPIE
IMPACT

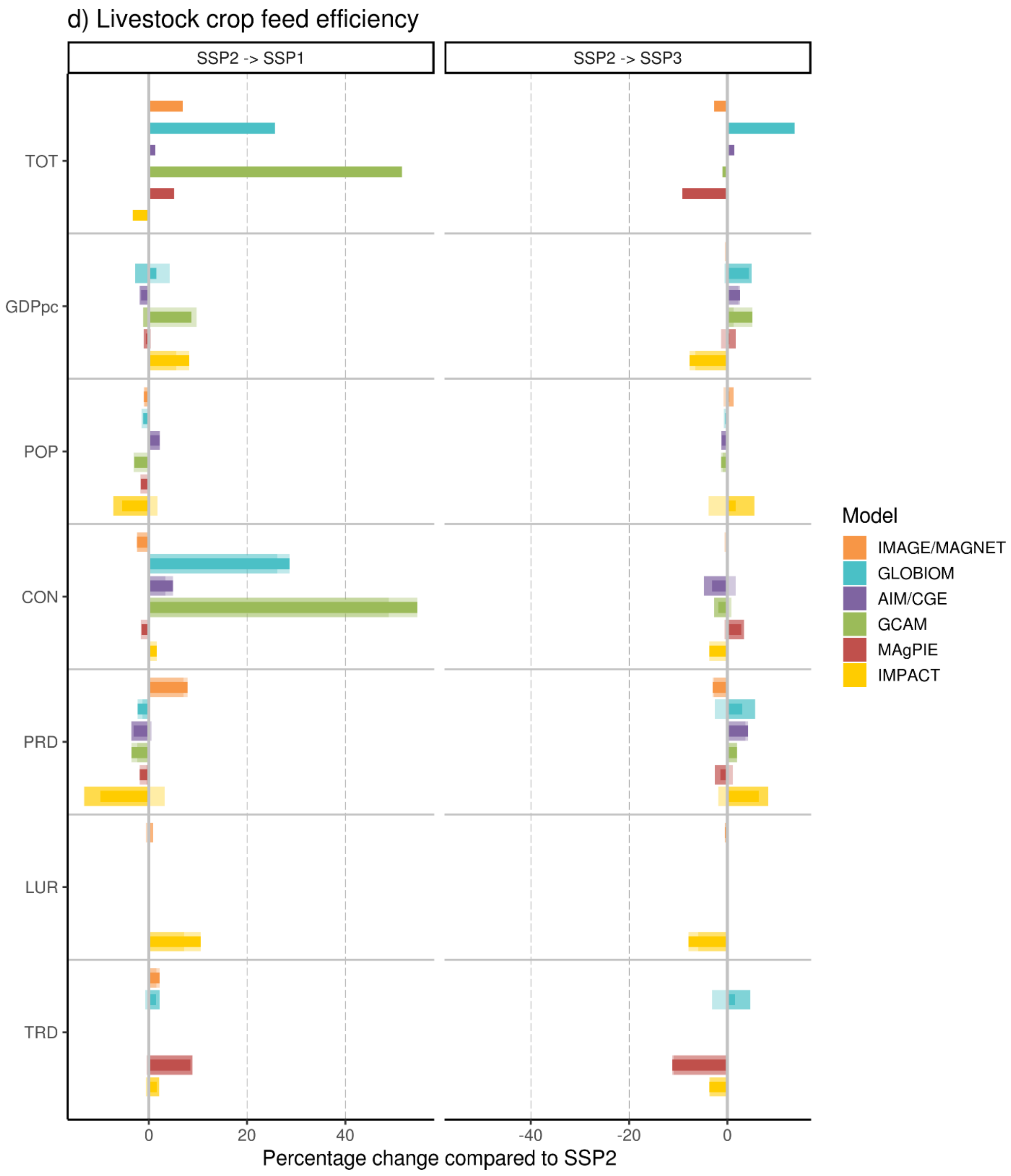

d) Livestock crop feed efficiency
SSP2 -> SSP1
SSP2 -> SSP3
TOT
GDPpc
POP
CON
PRD
LUR
TRD
0
20
40
-40
-20
0
Percentage change compared to SSP2
Model
IMAGE/MAGNET
GLOBIOM
AIM/CGE
GCAM
MAgPIE
IMPACT

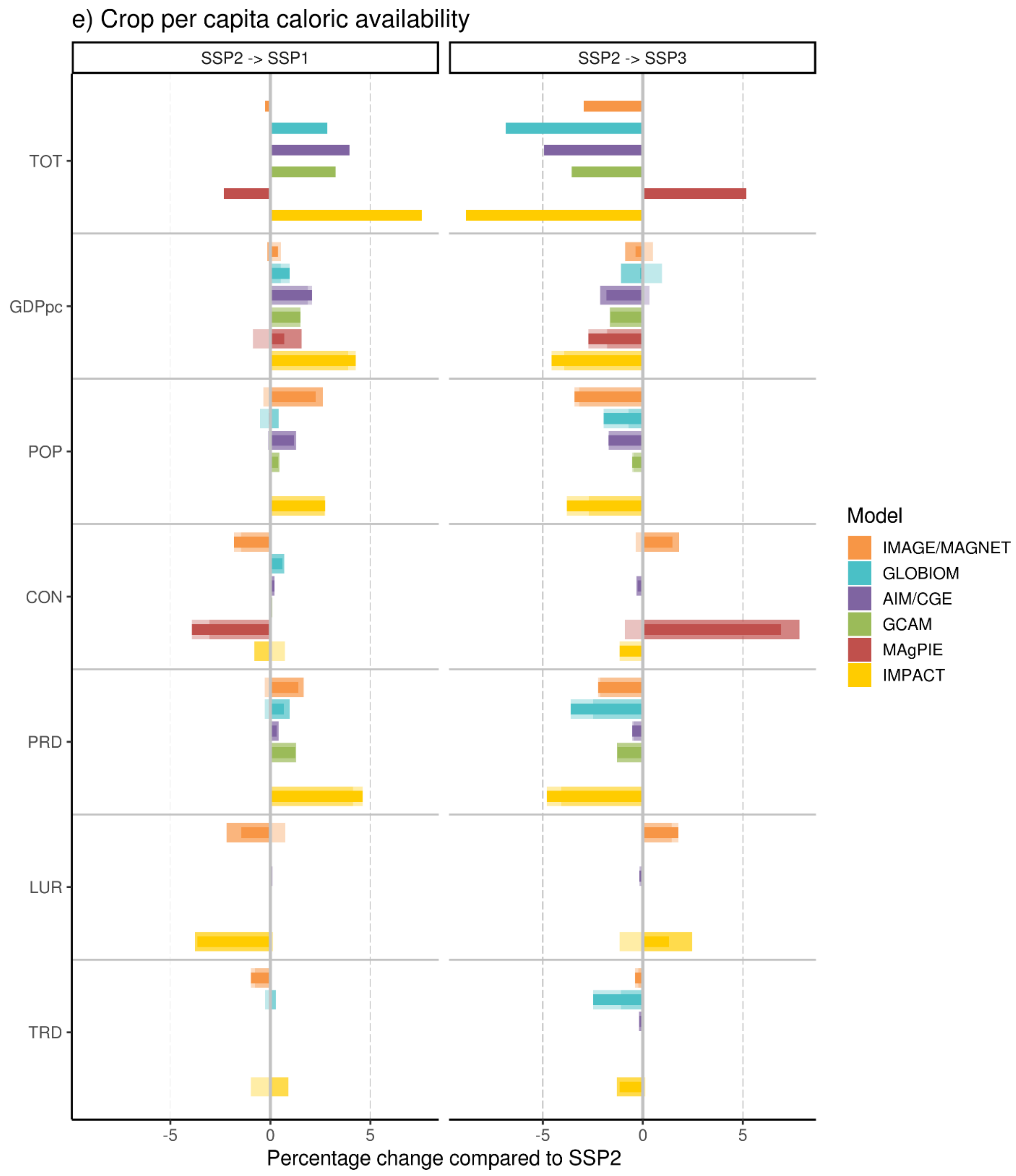

e) Crop per capita caloric availability
SSP2 -> SSP1
SSP2 -> SSP3
TOT
GDPpc
POP
CON
PRD
LUR
TRD
-5
0
5
Percentage change compared to SSP2
Model
IMAGE/MAGNET
GLOBIOM
AIM/CGE
GCAM
MAgPIE
IMPACT

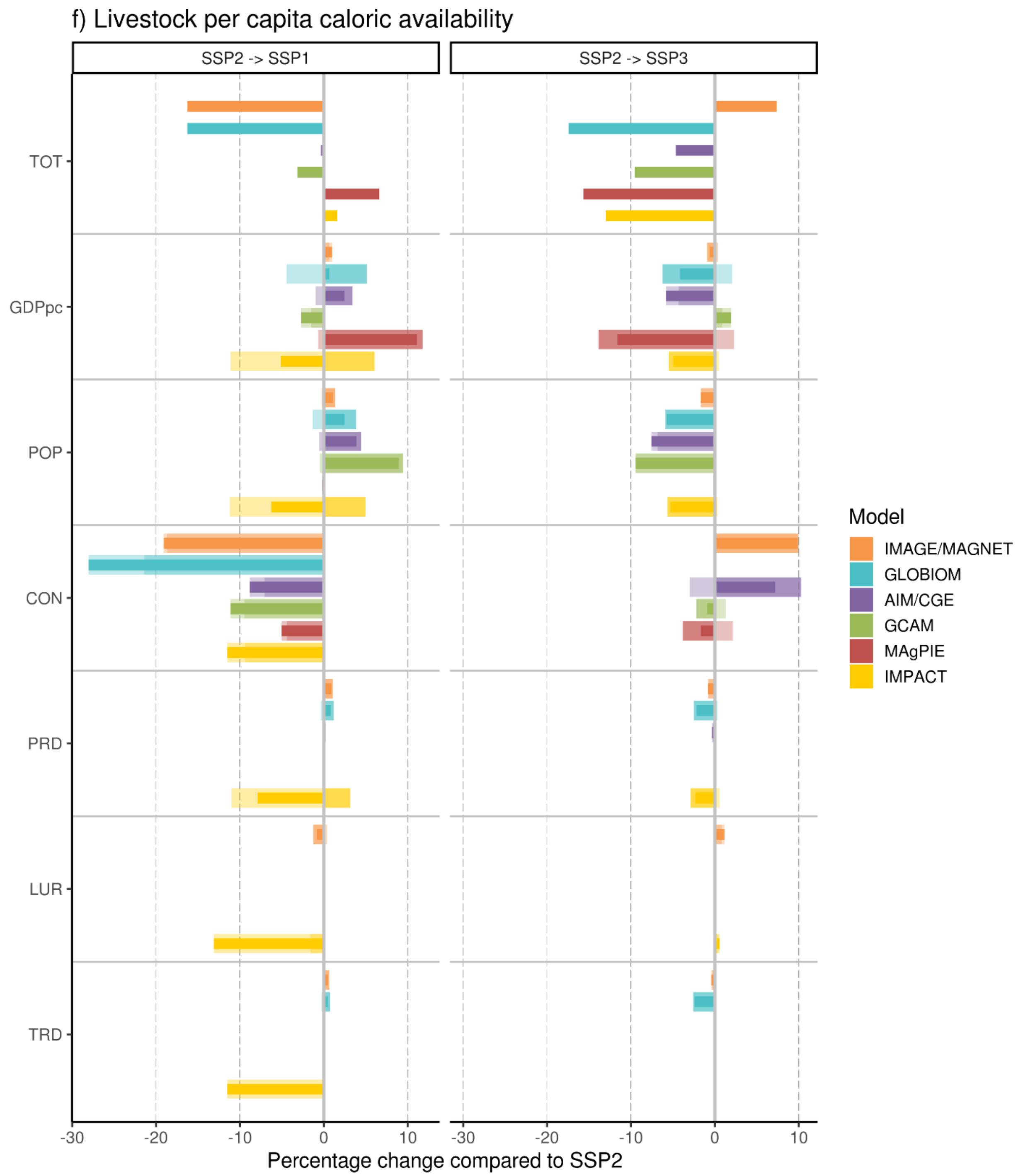

Supplementary Figure 5: Sensitivity of additional output variables of SSP1 and SSP3 to the six driver groups. Sensitivities reported for agricultural price (a), trade volumes (sum of net trade between AgMIP regions) (b), crop yields (c), livestock system efficiency (d), crop consumption (e), livestock consumption (f). Individual effects (wide, light colored bars), final effects (thin, dark colored bars) and interaction effects (wide, very light colored bars) are shown for each of the six driver groups (POP, GDPpc, LUR, PRD, CON, TRD), together with the total difference between SSP1 or SSP3, respectively, and SSP2 (TOT).

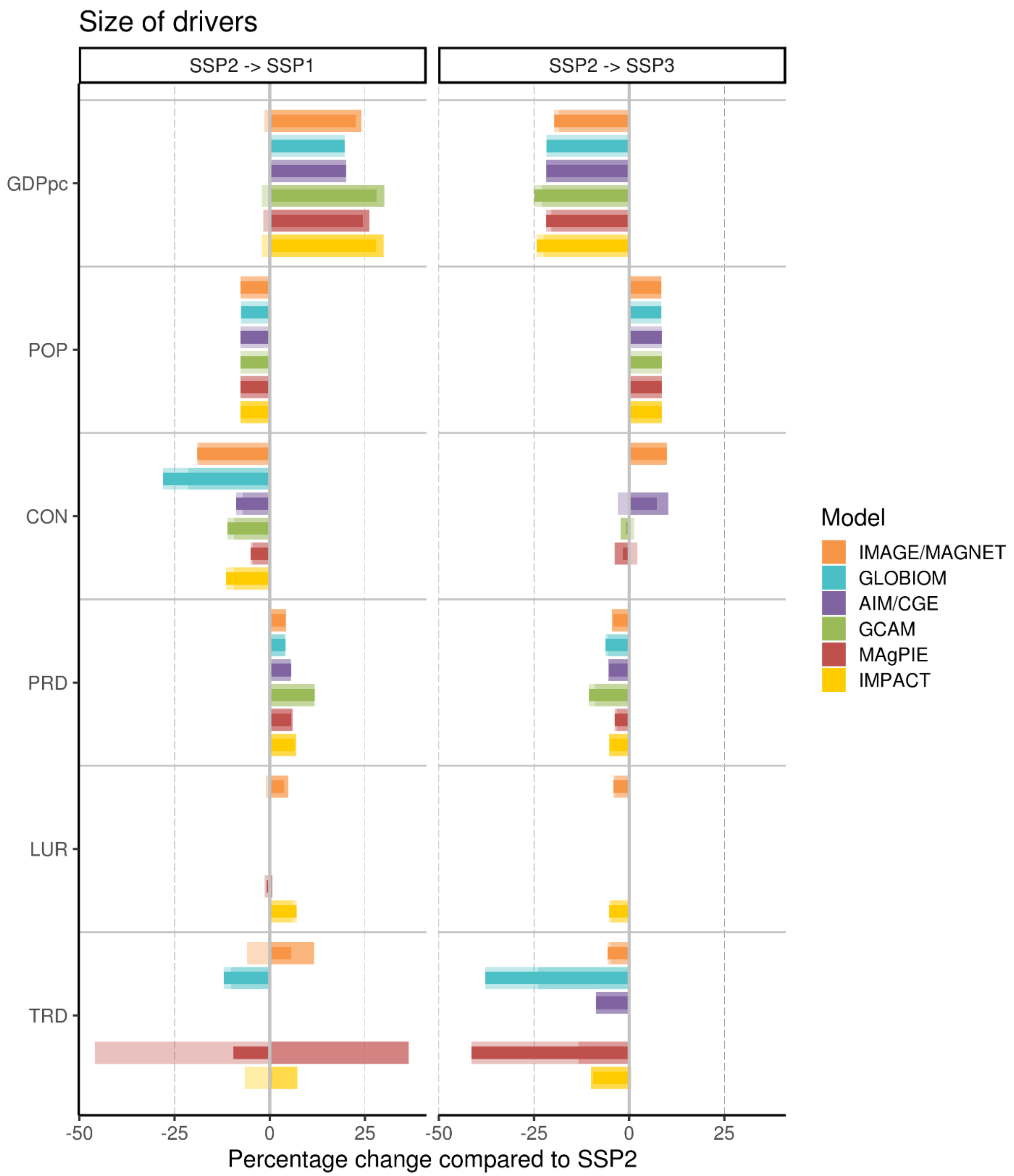

Supplementary Figure 6: Change of driving factors in SSP1 and SSP3 compared to SSP2. The size of the driver change was derived by measuring the change in the most proximate output variable, as shown in Supplementary Table 3.

a) Cropland area

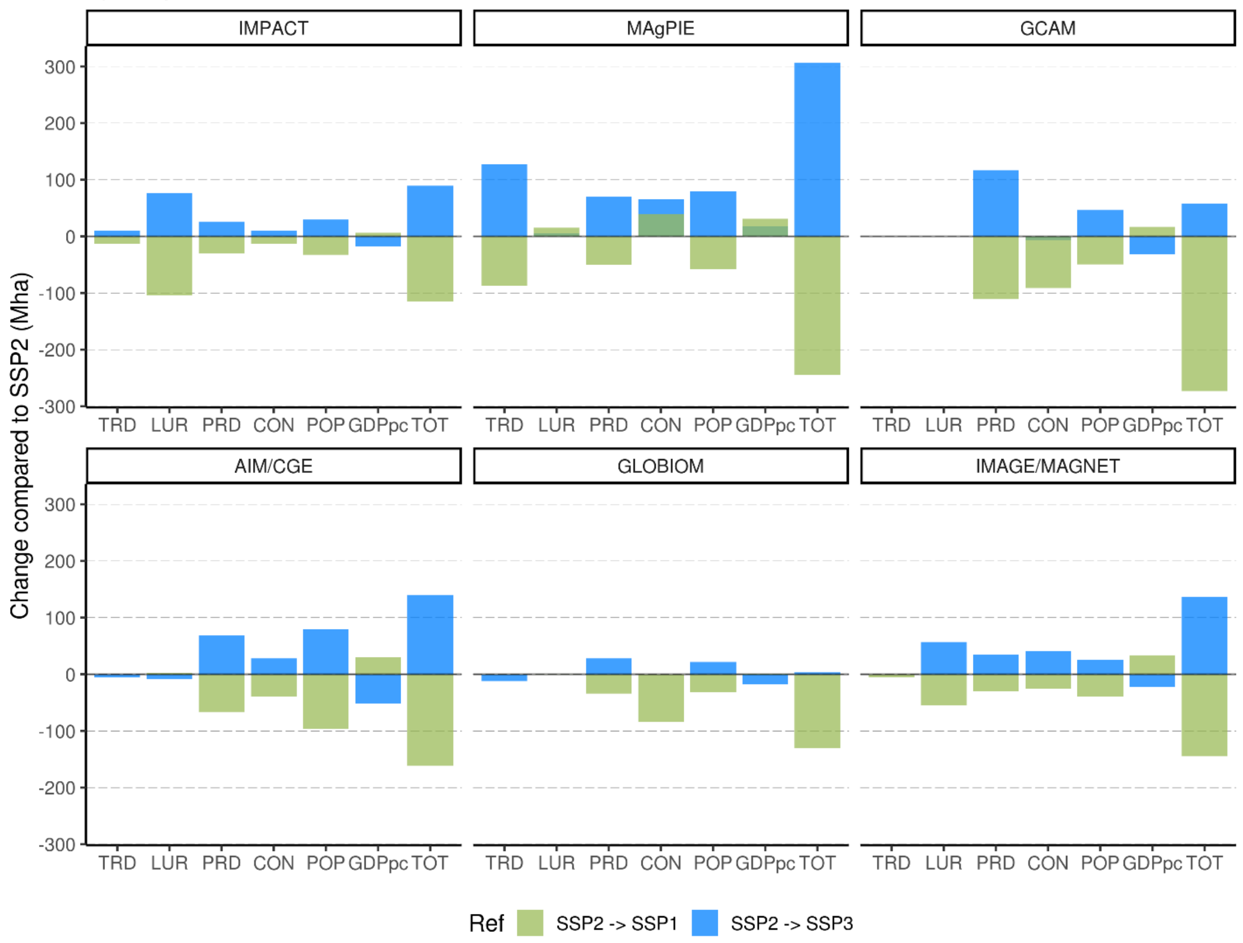

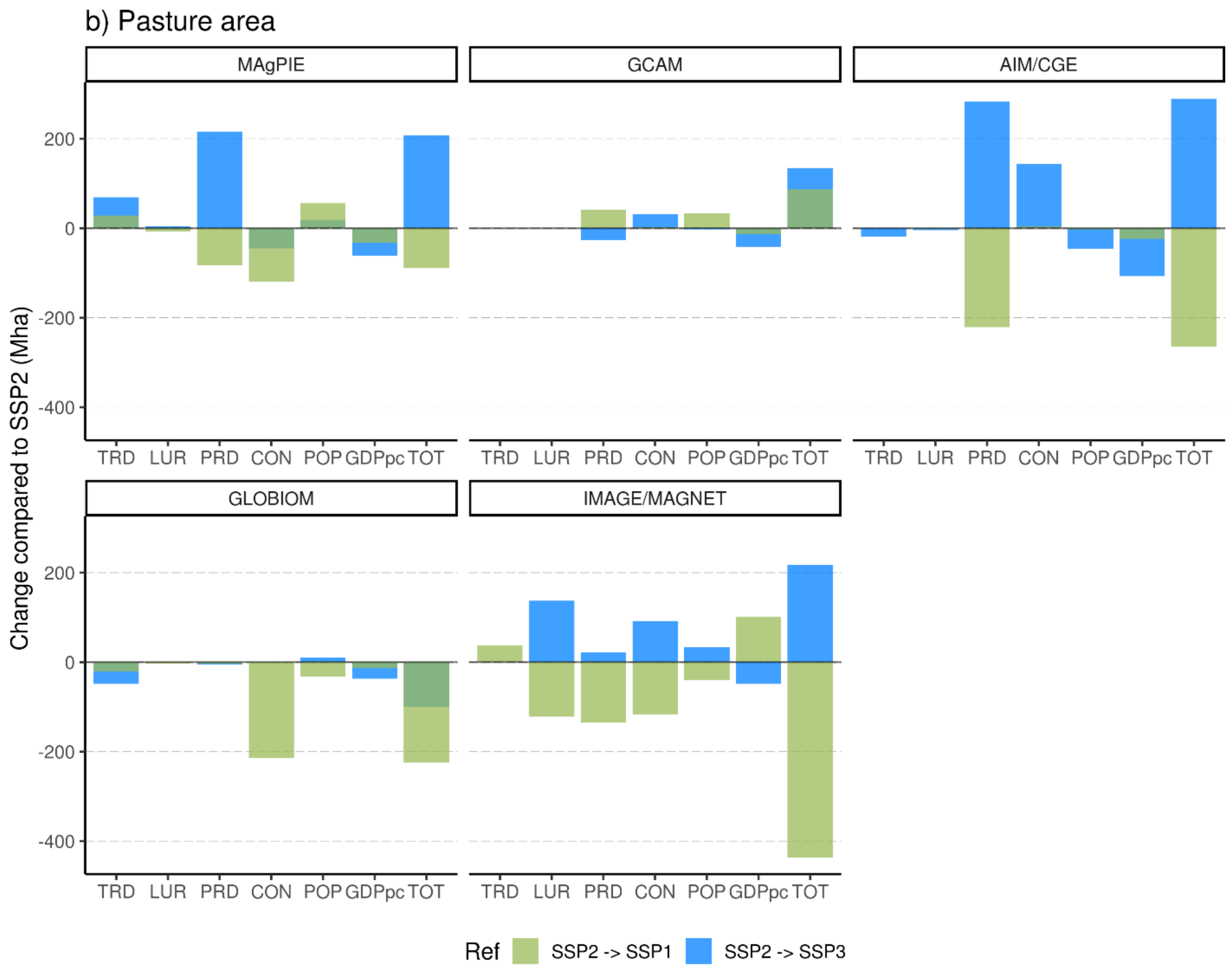

Supplementary Figure 7: Sensitivity in SSP1 and SSP3 scenarios to the six driver groups. Sensitivities of cropland area (a) and pasture area (b) are shown for SSP3 and SSP1 by driver group shown (average across individual and final effect), and total change, compared to SSP2.

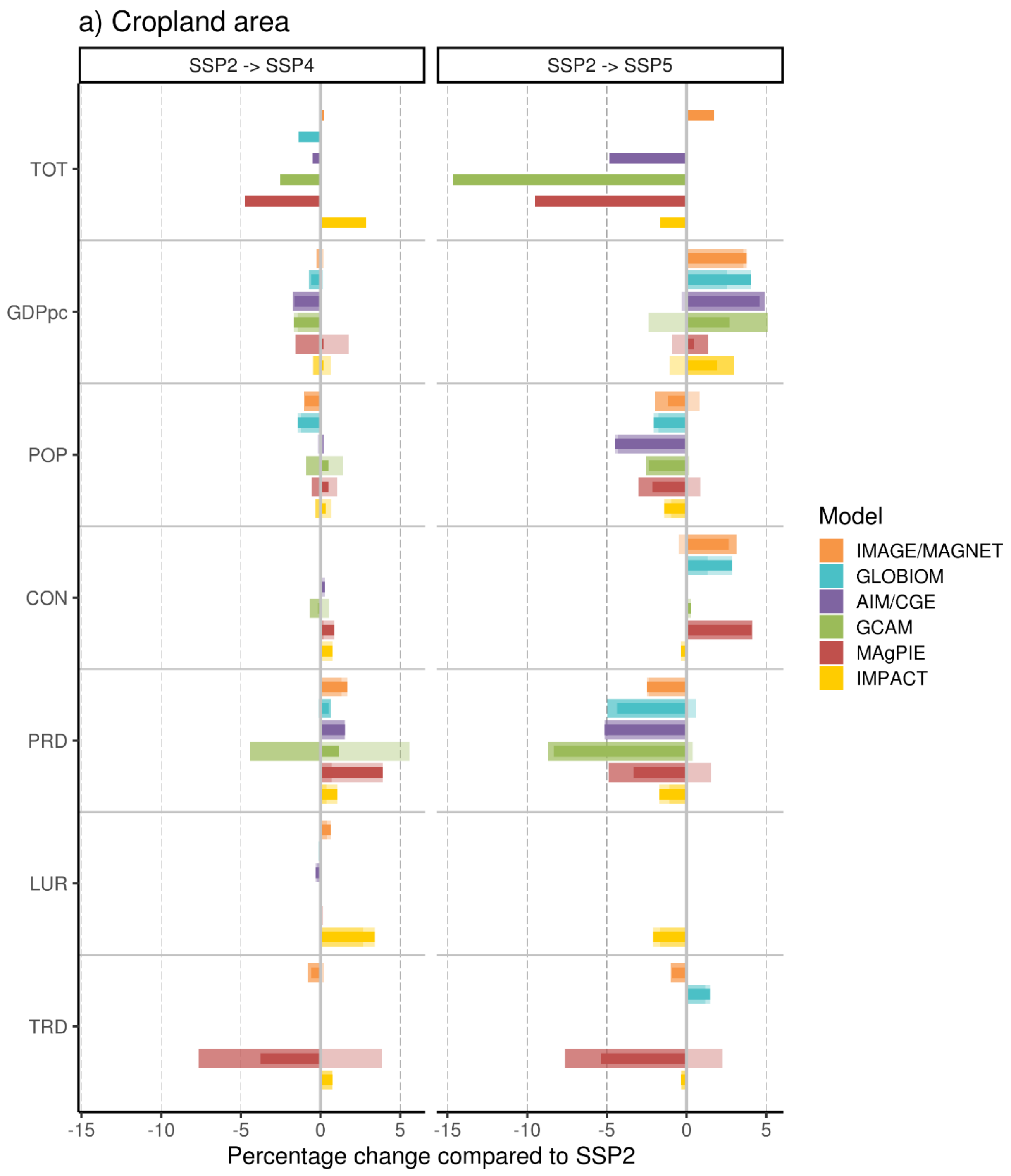
a) Cropland area
SSP2 -> SSP4
SSP2 -> SSP5
TOT
GDPpc
POP
CON
PRD
LUR
TRD
-15
-10
-5
0
5
Percentage change compared to SSP2
Model
IMAGE/MAGNET
GLOBIOM
AIM/CGE
GCAM
MAgPIE
IMPACT

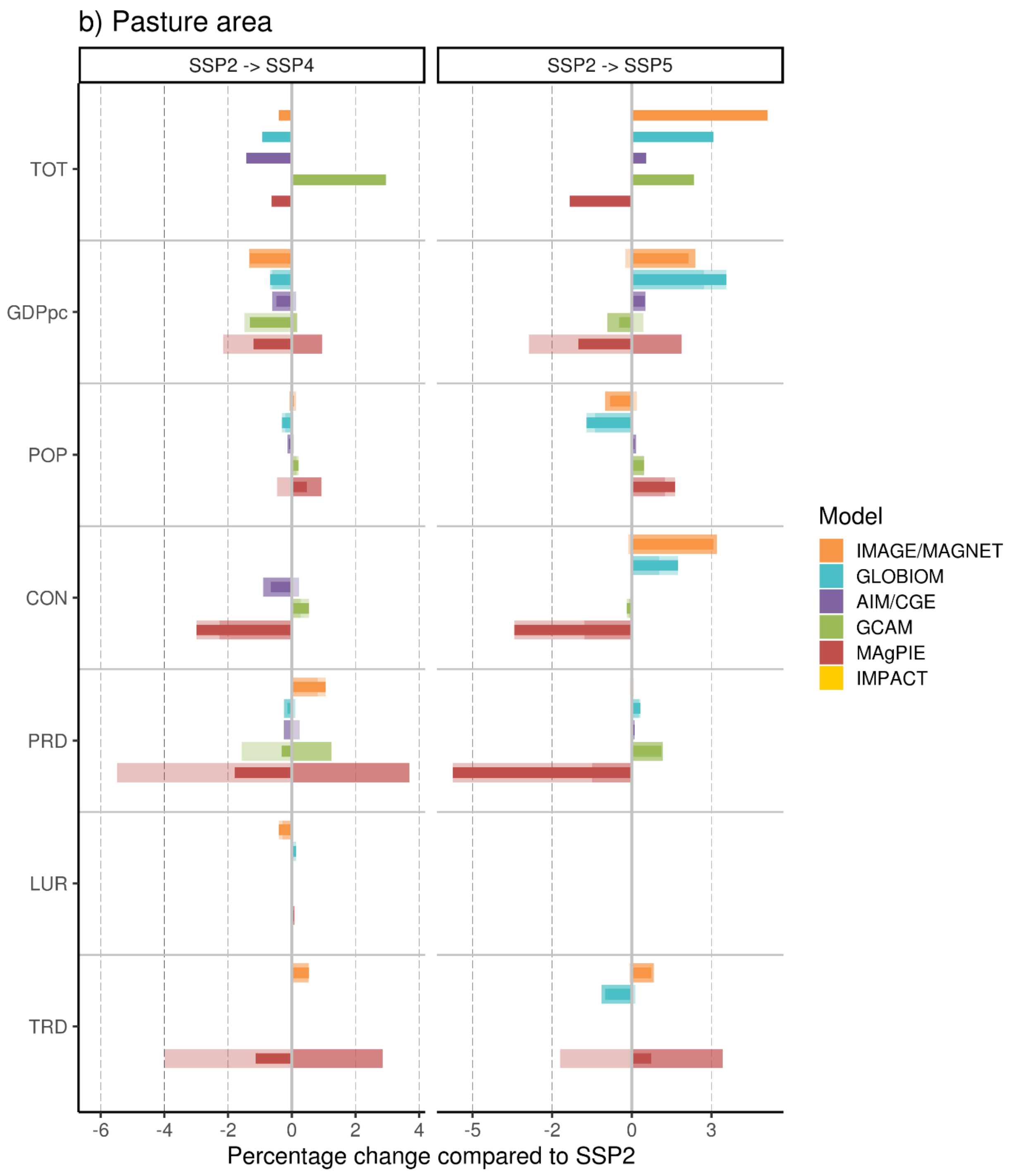

b) Pasture area
SSP2 -> SSP4
SSP2 -> SSP5
TOT
GDPpc
POP
CON
PRD
LUR
TRD
-6
-4
-2
0
2
4
-5
-2
0
3
Percentage change compared to SSP2
Model
IMAGE/MAGNET
GLOBIOM
AIM/CGE
GCAM
MAgPIE
IMPACT

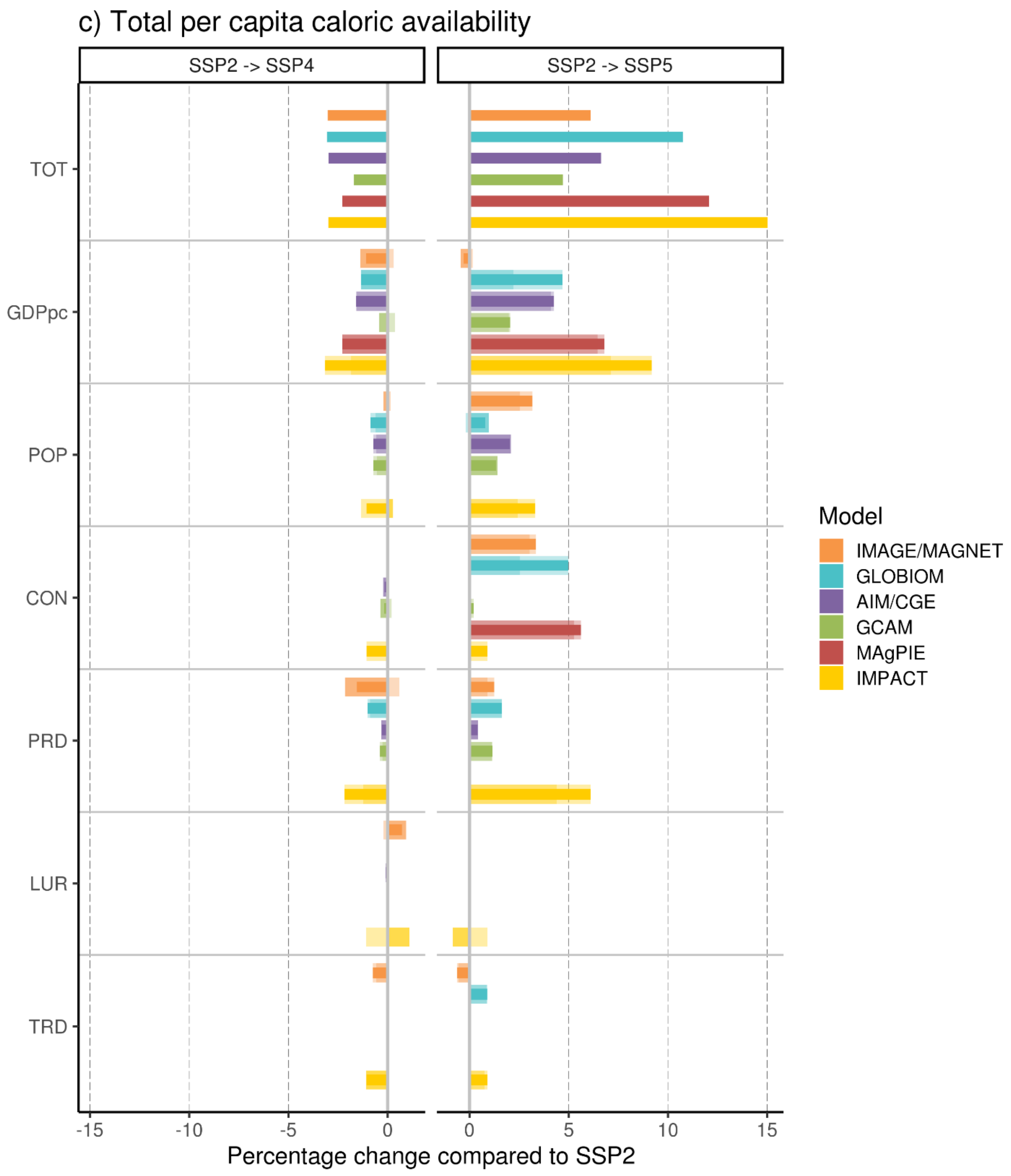
c) Total per capita caloric availability
SSP2 -> SSP4
SSP2 -> SSP5
TOT
GDPpc
POP
CON
PRD
LUR
TRD
-15
-10
-5
0
5
10
15
Percentage change compared to SSP2
Model
IMAGE/MAGNET
GLOBIOM
AIM/CGE
GCAM
MAgPIE
IMPACT

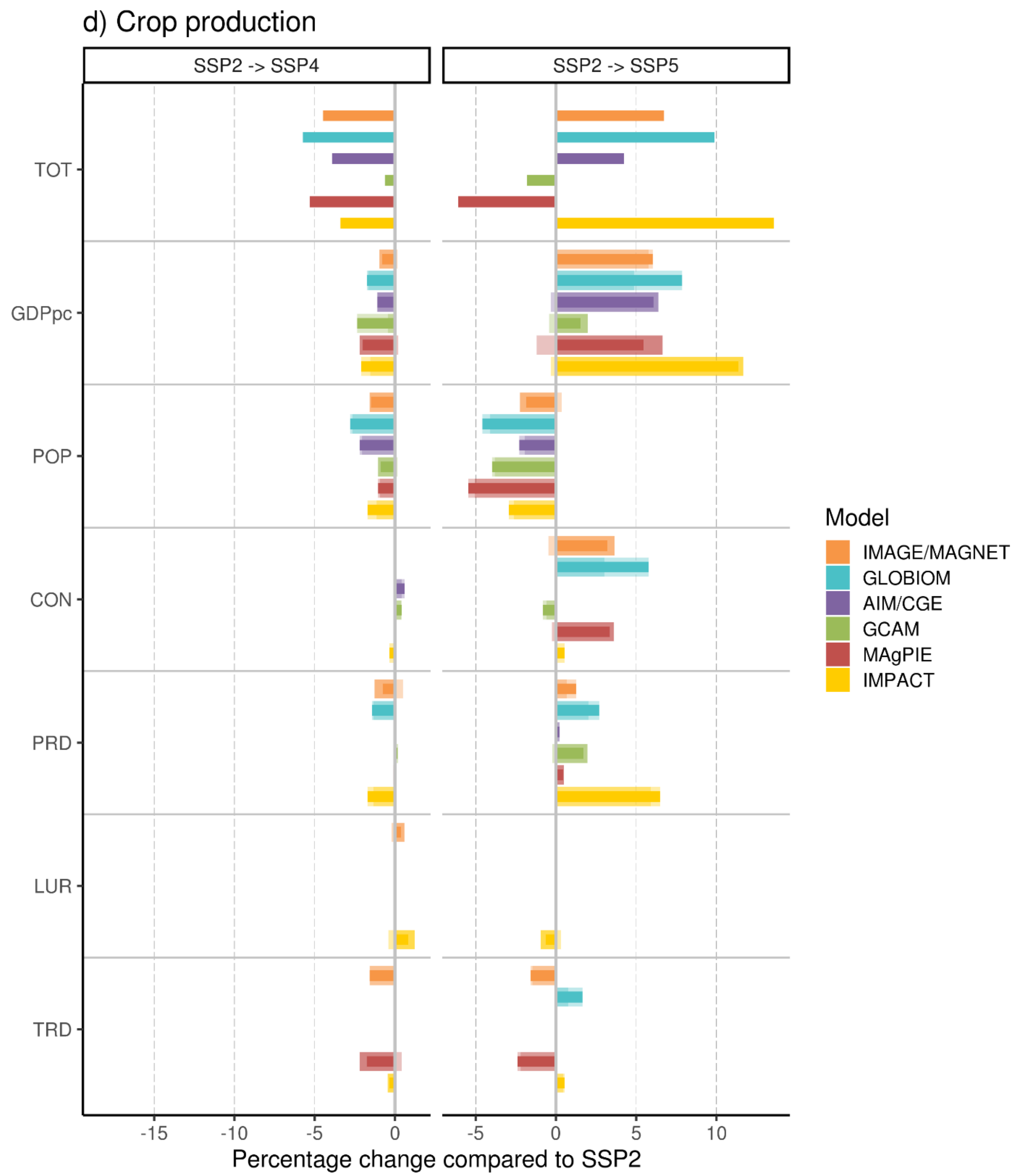

Supplementary Figure 8: Sensitivity of SSP4 and SSP5 to the six driver groups. Sensitivities reported for cropland (a), pasture (b), food demand (c), and crop production (d). Individual effects (wide, light colored bars), final effects (thin, dark colored bars) and interaction effects (wide, very light colored bars) are shown for each of the six driver groups (POP, GDPpc, LUR, PRD, CON, TRD), together with the total difference between SSP4 or SSP5, respectively, and SSP2 (TOT).

SSP	Description in Riahi et al. 2017
SSP1	SSP1 Sustainability – Taking the Green Road (Low challenges to mitigation and adaptation) The world shifts gradually, but pervasively, toward a more sustainable path, emphasizing more inclusive development that respects perceived environmental boundaries. Management of the global commons slowly improves, educational and health investments accelerate the demographic transition, and the emphasis on economic growth shifts toward a broader emphasis on human well-being. Driven by an increasing commitment to achieving development goals, inequality is reduced both across and within countries. Consumption is oriented toward low material growth and lower resource and energy intensity.
SSP2	SSP2 Middle of the Road (Medium challenges to mitigation and adaptation) The world follows a path in which social, economic, and technological trends do not shift markedly from historical patterns. Development and income growth proceeds unevenly, with some countries making relatively good progress while others fall short of expectations. Global and national institutions work toward but make slow progress in achieving sustainable development goals. Environmental systems experience degradation, although there are some improvements and overall the intensity of resource and energy use declines. Global population growth is moderate and levels off in the second half of the century. Income inequality persists or improves only slowly and challenges to reducing vulnerability to societal and environmental changes remain.
SSP3	SSP3 Regional Rivalry – A Rocky Road (High challenges to mitigation and adaptation) A resurgent nationalism, concerns about competitiveness and security, and regional conflicts push countries to increasingly focus on domestic or, at most, regional issues. Policies shift over time to become increasingly oriented toward national and regional security issues. Countries focus on achieving energy and food security goals within their own regions at the expense of broader-based development. Investments in education and technological development decline. Economic development is slow, consumption is material-intensive, and inequalities persist or worsen over time. Population growth is low in industrialized and high in developing countries. A low international priority for addressing environmental concerns leads to strong environmental degradation in some regions.
SSP4	SSP4 Inequality – A Road Divided (Low challenges to mitigation, high challenges to adaptation) Highly unequal investments in human capital, combined with increasing disparities in economic opportunity and political power, lead to increasing inequalities and stratification both across and within countries. Over time, a gap widens between an internationally-connected society that contributes to knowledge- and capital-intensive sectors of the global economy, and a fragmented collection of lower-income, poorly educated societies that work in a labor intensive, low-tech economy. Social cohesion degrades and conflict and unrest become increasingly common. Technology development is high in the high-tech economy and sectors. The globally connected energy sector diversifies, with investments in both carbon-intensive fuels like coal and unconventional oil, but also low-carbon energy sources. Environmental policies focus on local issues around middle and high income areas.
SSP5	SSP5 Fossil-fueled Development – Taking the Highway (High challenges to mitigation, low challenges to adaptation) This world places increasing faith in competitive markets, innovation and participatory societies to produce rapid technological progress and development of human capital as the path to sustainable development. Global markets are increasingly integrated. There are also strong investments in health, education, and institutions to enhance human and social capital. At the same time, the push for economic and social development is coupled with the exploitation of abundant fossil fuel resources and the adoption of resource and energy intensive lifestyles around the world. All these factors lead to rapid growth of the global economy, while global population peaks and declines in the 21st century. Local environmental problems like air pollution are successfully managed. There is faith in the ability to effectively manage social and ecological systems, including by geo-engineering if necessary.

Supplementary Table 1: SSP narratives [2,3]

Run #	Run name	Population (POP)	GDP per capita (GDPpc)	Land-use regulation (LUR)	Productivity growth (PRD)	Consumer preferences (CON)	Trade (TRD)
1	SSP2_BASE	2	2	2	2	2	2
2	SSP2_POP1	1	2	2	2	2	2
3	SSP2_GDP1	2	1	2	2	2	2
4	SSP2_LUR1	2	2	1	2	2	2
5	SSP2_PRD1	2	2	2	1	2	2
6	SSP2_CON1	2	2	2	2	1	2
7	SSP2_TRD1	2	2	2	2	2	1
8	SSP2_POP3	3	2	2	2	2	2
9	SSP2_GDP3	2	3	2	2	2	2
10	SSP2_LUR3	2	2	3	2	2	2
11	SSP2_PRD3	2	2	2	3	2	2
12	SSP2_CON3	2	2	2	2	3	2
13	SSP2_TRD3	2	2	2	2	2	3
14	SSP2_POP4	4	2	2	2	2	2
15	SSP2_GDP4	2	4	2	2	2	2
16	SSP2_LUR4	2	2	4	2	2	2
17	SSP2_PRD4	2	2	2	4	2	2
18	SSP2_CON4	2	2	2	2	4	2
19	SSP2_TRD4	2	2	2	2	2	4
20	SSP2_POP5	5	2	2	2	2	2
21	SSP2_GDP5	2	5	2	2	2	2
22	SSP2_LUR5	2	2	5	2	2	2
23	SSP2_PRD5	2	2	2	5	2	2
24	SSP2_CON5	2	2	2	2	5	2
25	SSP2_TRD5	2	2	2	2	2	5
26	SSP1_BASE	1	1	1	1	1	1
27	SSP1_POP2	2	1	1	1	1	1
28	SSP1_GDP2	1	2	1	1	1	1
29	SSP1_LUR2	1	1	2	1	1	1
30	SSP1_PRD2	1	1	1	2	1	1
31	SSP1_CON2	1	1	1	1	2	1
32	SSP1_TRD2	1	1	1	1	1	2
33	SSP3_BASE	3	3	3	3	3	3
34	SSP3_POP2	2	3	3	3	3	3
35	SSP3_GDP2	3	2	3	3	3	3
36	SSP3_LUR2	3	3	2	3	3	3
37	SSP3_PRD2	3	3	3	2	3	3
38	SSP3_CON2	3	3	3	3	2	3
39	SSP3_TRD2	3	3	3	3	3	2
40	SSP4_BASE	4	4	4	4	4	4
41	SSP4_POP2	2	4	4	4	4	4
42	SSP4_GDP2	4	2	4	4	4	4
43	SSP4_LUR2	4	4	2	4	4	4
44	SSP4_PRD2	4	4	4	2	4	4
45	SSP4_CON2	4	4	4	4	2	4
46	SSP4_TRD2	4	4	4	4	4	2
47	SSP5_BASE	5	5	5	5	5	5
48	SSP5_POP2	2	5	5	5	5	5
49	SSP5_GDP2	5	2	5	5	5	5
50	SSP5_LUR2	5	5	2	5	5	5
51	SSP5_PRD2	5	5	5	2	5	5
52	SSP5_CON2	5	5	5	5	2	5
53	SSP5_TRD2	5	5	5	5	5	2

Supplementary Table 2: Scenarios used in the sensitivity experiment. Numbers 1, 2, 3, 4 and 5 indicate from which SSP (1, 2, 3, 4, 5) the setting is applied.

Driver group	Proxy variable evaluated	Scenarios evaluated for individual effect	Comment
GDPpc	-		Available directly
POP			Available directly
CONX	a) Caloric food consumption of animal products, per capita	SSP2_CONX - SSP2	Relative change in proxy variable is used to estimate change in storyline driver compared to SSP2
	b) Total caloric food consumption, per capita	(alternative, not used)	
PRDX	a) Crop yield	SSP2_PRDX - SSP2	
	b) Livestock feed use efficiency	(alternative, not used)	
LURX	Agricultural area	SSP2_LURX - SSP2	
TRDX	Trade volume	SSP2_TRDX - SSP2	

Supplementary Table 3: Mapping of scenario drivers with proxy variables to derive the size of the drivers. To quantify the size of the driver groups (CON, PRD, LUR, TRD), proxy variables are compared between scenario SSPX (1, 3, 4, 5) and SSP2. The drivers GDPpc and POP are available directly in quantitative term. Results of this analysis shown in Supplementary Figure 6.

Supplementary References:

1 Marangoni, G. *et al.* Sensitivity of projected long-term CO_2 emissions across the Shared Socioeconomic Pathways. *Nature Climate Change* **7**, 113 (2017).

2 Riahi, K. *et al.* The Shared Socioeconomic Pathways and their energy, land use, and greenhouse gas emissions implications: An overview. *Global Environmental Change* **42**, 153-168, doi:10.1016/j.gloenvcha.2016.05.009 (2017).

3 O'Neill, B. C. *et al.* The roads ahead: narratives for shared socioeconomic pathways describing world futures in the 21st century. *Global Environmental Change* **42**, 169-180 (2017).
