# Supplementary material for: Key determinants of global land-use projections
Source: Nat Commun. 2019 May 15;10:2166. doi: 10.1038/s41467-019-09945-w (PMC6520344; doi:10.1038/s41467-019-09945-w)
Supplement: Supplementary file 3 — Description of Additional Supplementary Files [file 41467_2019_9945_MOESM3_ESM.pdf]

Description of Additional Supplementary Information

File Name: Supplementary Data 1

Description: Excel table on the implementation of scenario drivers, by model.
